# Cutaneous Thermography in Arthropathies: Quantitative Imaging, Machine Learning, and Clinical Translation

**DOI:** 10.3390/jimaging12060270

**Published:** 2026-06-18

**Authors:** Constantin-Adrian Andrei, Serban Dragosloveanu, Alex-Gabriel Grigore, Andreea Alexandra Anghel, Atanasie-Andrei Gogu, Rares-Mircea Birlutiu, Christiana Diana Maria Dragosloveanu, Catalin Anghel, Adrian Iftime, Romica Cergan, Constantin Caruntu, Cristian Scheau

**Affiliations:** 1 Faculty of Medicine, The “Carol Davila” University of Medicine and Pharmacy, 050474 Bucharest, Romania; 2 “Foisor” Clinical Hospital of Orthopaedics, Traumatology and Osteoarticular TB, 021382 Bucharest, Romania; 3 Faculty of Automation, Computer Science, Electrical and Electronic Engineering, “Dunărea de Jos” University of Galati, 800008 Galati, Romania; 4 Faculty of Dentistry, The “Carol Davila” University of Medicine and Pharmacy, 041292 Bucharest, Romania; 5 Department of Ophthalmology, Clinical Hospital for Ophthalmological Emergencies, 010464 Bucharest, Romania; 6 Department of Computer Science and Information Technology, “Dunărea de Jos” University of Galati, 800146 Galati, Romania; 7 Department of Dermatology, “Prof. N.C. Paulescu” National Institute of Diabetes, Nutrition and Metabolic Diseases, 011233 Bucharest, Romania

**Keywords:** infrared thermography, arthropathy, artificial intelligence, machine learning, region of interest

## Abstract

Arthropathies are a major global health challenge because of their high prevalence, chronic progression, and significant impact on quality of life and health systems. Therefore, prompt and accurate diagnosis is critical for slowing disease progression and improving outcomes. Traditional imaging modalities, such as ultrasound and magnetic resonance imaging, suffer from significant limitations, including operator dependence, limited accessibility, high cost, and limited reproducibility. Infrared thermography has become a promising non-invasive imaging technique for identifying thermal variations linked to inflammatory and metabolic processes. Advances in quantitative thermography, automated segmentation, and artificial intelligence have greatly enhanced its clinical applicability. This review summarizes recent advances in thermography-based biomarkers, including region-of-interest-derived metrics, asymmetry indices, hotspot burden, spatial and texture descriptors, and composite thermographic scores. It discusses the role of machine learning and deep learning in prediction, phenotyping, and multimodal integration with clinical, laboratory, and imaging data. Heterogeneity of protocols, variability in measurements, domain shift, validation design, overfitting, and reporting quality are also addressed. Overall, thermography combined with AI is highly promising as an adjunct to early diagnosis, assessment of disease activity, and follow-up in arthropathies. However, clinical application at a large scale requires strict standardization, external validation, transparent reporting, and well-elucidated, reproducible analytical processes.

## 1. Introduction

Rheumatic and musculoskeletal diseases (RMDs), including many inflammatory and degenerative arthropathies, constitute a substantial global health concern due to their high prevalence, chronic progression, and considerable effects on both patient quality of life and medical care systems [1]. Rheumatoid arthritis (RA) affects approximately 0.24% to 1% of the global population [2]. Effective management of such conditions often requires long-term, intensive treatment [3]. Early and precise diagnosis is critical to slowing disease progression [4] and improving patient outcomes, given that metabolic and tissue alterations may occur before discernible inflammation [5,6,7]. Standard diagnostic techniques frequently exhibit inadequate sensitivity and specificity, particularly during the initial phases of disease [6]. Clinically, magnetic resonance imaging is deemed expensive and unsuitable for repeated use [8], while ultrasound is operator-dependent and subject to reproducibility concerns despite improvements through standardized scoring systems [9], thereby increasing the risk of irreversible joint damage. Because of that, new diagnostic methods have been sought over several decades to achieve faster, more reliable, and reproducible results in assessing early-onset arthropathies.

Infrared thermography is an emerging noninvasive imaging modality for assessing musculoskeletal disorders [10]. This method can distinguish small temperature changes related to joint inflammation as a result of changes in blood flow [11]. As inflamed or metabolically active tissues display elevated skin temperatures due to elevated blood flow and cellular activity [12], thermal imaging can differentiate between healthy joints and those affected by various arthropathies [13].

Infrared thermography provides a unique view of arthropathic pathology by combining functional data with structural imaging. Recent studies show that it works as a simple, accurate, and noninvasive tool for screening and monitoring rheumatoid arthritis and osteoarthritis [13]. Historically, the interpretation of thermographic data often used simple qualitative or quantitative measures that did not always account for the complexity and variability of thermal patterns in arthropathies [13]. New quantitative imaging and machine learning methods have been developed to extract more accurate, detailed information from thermographic data to address this issue [14]. These methods allow observations of all the spatial and temporal thermal patterns associated with disease activity [15]. Recent advances in quantitative imaging and machine learning, especially deep learning for automatic image segmentation and feature extraction, have significantly improved the ability of infrared thermography to interpret data [16]. By linking thermal signatures to clinical activity indices, AI applications in thermal data analysis provide new insights into disease mechanisms and treatment responses [15]. Therefore, this method may be a valuable tool for helping doctors diagnose diseases early, predict outcomes, and monitor arthropathies.

**Figure 1:**
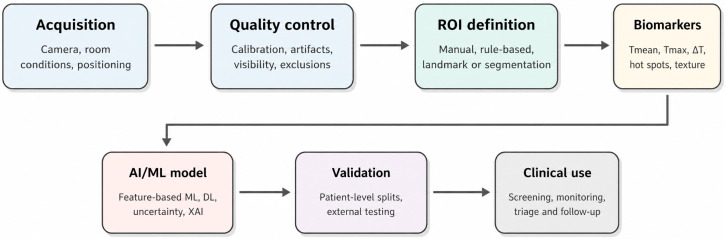
Unified translational workflow linking thermographic acquisition, ROI definition, quantitative biomarkers, AI/ML modelling, validation, and clinical deployment in arthropathy assessment.

In this article, we review and critically integrate the recent technological developments in quantitative infrared thermography and machine learning for diagnosis, phenotyping, monitoring, and clinical implementation of arthropathies. Although recent reviews have addressed the clinical applicability, diagnostic potential, and disease-specific evidence for infrared thermography in rheumatic and degenerative joint diseases [11,17,18,19,20,21,22,23,24,25,26], they have generally provided limited integration of quantitative image analysis, machine learning/deep learning workflows, validation strategies, dataset heterogeneity, and clinical translation [27]. Thus, this review focuses specifically on the quantitative and translational pipeline that enables thermography to move beyond simply descriptive thermal imaging, into a tool to support clinical decision-making. Rather than evaluating studies individually, this review examines the literature through a unified framework comprising six interconnected components (Figure 1): acquisition protocol, ROI definition, extraction of thermographic biomarkers, modeling based on AI/machine learning (ML), validation procedure, and clinical utilization. This is necessary, because several discrepancies in the literature do not originate from thermography itself, but from differences in the type of camera, camera acquisition environment, definition of anatomical regions of interest, reference criteria, building of models, and experimental validation. This fact requires evaluation of the clinical utility of thermography based not only on the specific values of diagnostic performance but primarily on its repeatability and external validity, and on the actionability of the entire workflow in a clinical setting. Therefore, in this article we review ROI-derived metrics, asymmetry indices, hotspot burden, automatic ROI identification, machine and deep learning methodologies, thermographic composite scores for disease activity, and validation requirements. We analyze in depth the specific technological issues for clinical implementation, including camera system variability and the ensuing domain-shift, feature selection, multicollinearity, external validation of the models, quality of reporting and confidence intervals, classification of regulatory issues, and integration in the clinical workflow. In this way, while this review departs from previous studies centered on a specific disease, we focus on the development of thermography as a quantitative imaging tool enabled by AI.

## 2. Quantitative Thermography and AI-Driven Analytics

Quantitative infrared thermography (IRT) is evolving from basic “hot/cold spot” inspections, which merely provide a general sense of temperature, to more precise measurements that can be repeatedly conducted. This change is necessary to reduce the risk of observer bias, improve long-term objective monitoring, and better integrate the test with other rheumatology and orthopedic tests. When acquisition conditions remain stable, quantitative IRT can employ region-of-interest (ROI) descriptors, including mean and maximum temperature, along with distributional statistics. Additionally, it can utilize contrast indices, such as intra-ROI gradients or side-to-side ΔT, to characterize localized thermal patterns. This provides us with brief indicators that can be utilized to compare visits and joints [10,17].

Concurrently, quantitative infrared thermography is gaining popularity, as advancements in computer image analytics facilitate greater scalability and repeatability. Automating the construction and segmentation of ROIs can significantly reduce the errors that often occur when performed manually. Machine learning algorithms can transform various thermal descriptors into clinically relevant outputs, such as the likelihood of inflammation or activity, indicators of severity, and triage ratings [28]. When evaluating an individual’s work, it is essential to consider the validation design, the reporting of protocols, and any additional factors that might independently influence skin temperature. Several factors may influence skin temperature independently of joint pathology [16,29].

The most dominant finding from this literature review is that no quantitative evaluation of thermal data can be conducted in isolation from the processes by which the data are acquired. Temperature within the ROI, asymmetry scores, burden of hotspots, and various textural measures of the ROI all seem to make clinical sense, yet cannot be reliably compared across different research studies in which image acquisition protocols, ROI definition, thermal references, and endpoints differ [30]. Perhaps the most telling is the observation that those studies offering the best classification rates are often not those with well-defined protocols for acquisition and analysis [31]. Therefore, throughout this review, the thermographic metrics are presented as part of a larger measurement pipeline, not as standalone disease biomarkers, a framing that permits identification of true disease markers, distinct from artifacts introduced by the image acquisition protocol, camera quality, or over-training of algorithms [32].

### 2.1. ROI-Based Quantitative Metrics

Utilizing ROI in quantitative analysis is the most direct approach for transforming infrared thermography (IRT) into clinically applicable metrics. ROI measurements clearly demonstrate local hyperthermia by depicting the temperature distribution within anatomically defined areas. When acquisition and ROI placement are standardized, these parameters can be longitudinally evaluated and compared among patients. Standard metrics encompass measurements of central tendency and extremes (e.g., mean and high-percentile temperatures), dispersion within the ROI, and differential indices such as side-to-side ΔT or variations between joint and neighboring tissues, which can partially control for inter-individual baseline variability and systemic influences [33,34]. However, the diagnostic application of ROI indicators relies on a defined definition of ROI, meticulous selection of benchmarks, and clear specification of the selected summary statistics and thresholds [35,36].

### 2.2. ROI Definition and Reference Strategies

Uniform and anatomically accurate delineation of the ROI is essential for reliable ROI-based thermographic evaluations. In musculoskeletal research, ROIs should be precisely centered on the joint line and adjacent tissues to enable consistent comparisons across visits and between patients. Selecting ROIs solely based on maximal intensity is inadequate. The use of standardized acquisition protocols and explicitly defined ROI geometry, including specific dimensions, shapes, and anatomical landmarks, reduces operator-dependent variability and improves the reproducibility of results [35].

Choosing a suitable standard for evaluating ROI metrics is equally essential. Common strategies encompass: (i) analyzing the left and right sides (left–right ΔT) when one side is minimally modified and functions as an internal control; (ii) employing a local reference ROI in adjacent tissue (joint ROI minus nearby non-joint area) to alleviate global thermal fluctuations; and (iii) establishing within-patient longitudinal baselines, such as alterations from previous visits, to evaluate treatment efficacy. Clearly defining these reference alternatives is crucial, as they directly affect impact sizes and the assessment of research outcomes [36].

Diverse methodologies produce the most significant outcomes when normative benchmarks and acquisition parameters are well delineated. Extensive datasets from healthy persons establish reference standards for limb surface temperatures and enable comparative analyses of infrared thermography (IRT) in joint-related diseases [37].

### 2.3. Core Temperature Statistics Within the ROI

Once a ROI is defined, quantitative infrared thermography (IRT) is typically operationalized through descriptive summaries of the ROI temperature distribution. These statistics provide a clinically interpretable representation of periarticular thermal status and facilitate longitudinal assessment, provided that acquisition conditions and ROI placement are sufficiently standardized [36].

Numerous studies indicate at least one measure of central tendency, such as the mean (Tmean) or median (Tmedian) temperature of the ROI. Tmean is often used to represent the average temperature around a joint, but Tmedian might be better when the temperature data is uneven or has unusual values. Descriptions of the upper tail offer supplementary insights that more precisely represent focal hyperthermia. The maximum temperature (Tmax) is intuitive but very susceptible to outliers and edge effects. Consequently, some quantitative methodologies employ elevated percentiles (such as P90 or P95) to more effectively delineate the top tail. Dispersion measures, including standard deviation (SD) and interquartile range (IQR), are frequently presented simultaneously to assess the uniformity or heterogeneity of heating within the ROI [36].

In knee arthritis, for example, ROI-level summaries, including Tmax, Tmin, and average temperature have been extracted from thermograms and compared across clinically and imaging-defined groups, illustrating the use of simple ROI descriptors as quantitative markers in musculoskeletal cohorts [34]. For consistency across studies, a practical approach in review writing is to ensure that at least one central tendency metric (Tmean or Tmedian), one upper-tail descriptor (P95 or Tmax), and one dispersion metric (SD or IQR) are explicitly reported for each ROI [36].

### 2.4. Differential and Asymmetry Metrics (ΔT-Based Indices)

Differential measures address inter-individual variability by utilizing within-subject differences instead of absolute ROI temperatures. Contralateral asymmetry is the most straightforward method to achieve this. The temperature differential between analogous regions of interest on the left and right sides (ΔT_L–R). This technique employs physiological thermal symmetry to facilitate the identification of localized inflammation when one side is less impacted. However, its reliability diminishes in the presence of illness on both sides, a prior injury, or significant side dominance [18].

A second set of indices uses local reference contrasts to account for global thermal changes, such as changes in the environment or generalized vasomotor changes. In this case, ΔT is calculated between the joint ROI and a nearby reference region that is thought to be less involved (for example, the difference in temperature between the knee and thigh), which makes periarticular heat stand out compared to nearby tissue. Localized ΔT metrics have been shown to be clinically relevant in RA because knee-thigh temperature differences can tell the difference between inflamed and control knees and are linked to ultrasound power Doppler findings [38].

In more recent times, algorithmic formulations have made this principle more formal by using within-limb calibration to define ΔT as the difference between a joint ROI and an ipsilateral reference site (such as the mid-tibia). Both mean and upper-tail (such as the 95th percentile) calibrated measures are then used as diagnostic features. Researchers have found that using within-limb calibrated metrics instead of absolute temperatures can help tell the difference between different types of pediatric arthritis. This shows how reference-based ΔT indices can reduce variability caused by the baseline and the device [39].

When applied to arthropathies, the significance of the thermal difference ΔT will depend on the predicted pattern of joint distribution. Given the largely bilateral and somewhat symmetric involvement in RA, simple contralateral comparison of ΔT has limited value, especially when the same joints are affected on the homologous side, in which case a reference region and comparison with ultrasound-detected synovitis is better. If one joint, especially for knee OA with symptoms, has noticeably greater symptoms than the opposite joint, then the comparison of ΔT will provide valuable information regarding localized and unilateral inflammation. The use of ΔT for Juvenile Idiopathic Arthritis and other pediatric arthropathies is tempting, as it is non-invasive, but consideration of patient age, the influence of anatomy upon development, joint size and position, and the use of age-matched reference regions is required during analysis. In arthropathies such as psoriatic arthritis, gout, septic arthritis, or inflammatory conditions following injury, ΔT has little use apart from showing local inflammation or vascular activity. It lacks the specificity to be of any use without clinical correlation and imaging findings.

### 2.5. Intra-ROI Heterogeneity and Gradient Measures

In many arthropathies, periarticular heating is not spatially uniform; therefore, a useful addition is to look at the temperature changes within the same ROI using simple measures, including standard deviation or interquartile range, and contrast measures such as the difference between the highest and lowest temperatures. These measures help enhance average temperature readings by separating general heat from specific “hot” spots, especially when inflammation is occasional or limited to certain parts of a joint [40].

Gradient-oriented methods can also be used to describe spatial non-uniformity, either implicitly (by comparing preset sub-ROIs within the same joint field) or explicitly (by looking at temperature differences between nearby zones). Subregional mapping is particularly beneficial in extensive joints where several compartments may display unique heat patterns. In symptomatic knee osteoarthritis, taking temperatures from several standardized knee sub-areas (such as the medial, lateral, patellar, and suprapatellar regions) has been used to show clinically significant differences across the joint surface. This supports the idea that thermographic changes related to arthropathy may show up as regional patterns instead of uniform shifts [41].

Finally, pattern-aware analytics are increasingly incorporating heterogeneity information at scale by aggregating measurements across multiple joint sites and/or utilizing learned indices. Recent longitudinal studies found that composite thermographic summaries (incorporating Tmax-, Tavg-, and Tmin-based aggregates across prevalent joint sites) can detect subclinical synovitis in rheumatoid arthritis, on par with ultrasound diagnostic. Doppler inflammation severity and externally validated machine learning-based thermographic indices have been assessed as quantitative indicators of joint inflammation and disease activity [15].

### 2.6. Hotspot Burden and Area-Based Metrics

Besides single-point or summary temperatures (e.g., Tmax, Tmean), the geographical distribution of hyperthermia can yield therapeutically pertinent information, especially when inflammation is localized or restricted to a joint area. “Hot-spot burden” assesses the extent of exceptional warmth within the ROI, rather than merely identifying the temperature of the hottest pixel.

The first step is to use thresholding or isothermal segmentation to mark off a hot area. Thresholds can be (i) relative to a reference, such as a contralateral joint, adjacent tissue, or the patient’s baseline, or (ii) established by distribution, such as pixels exceeding a specified percentile in the area of interest. Relative definitions are usually more useful in a wider range of clinical situations than absolute cutoffs, which can be affected by things like the weather, skin emissivity assumptions, and camera calibration.

Once a hot region is defined, the most common area-based endpoints include:

- Hotspot area (HSA): absolute area (cm^2^) or pixel count above the threshold.
- Hotspot fraction: HSA divided by total ROI area (%), improving comparability across ROIs of different sizes.
- Hotspot count: the number of disconnected hot clusters (useful when inflammation is multifocal).
- Thermal burden indices that combine temperature elevation and spatial extent. A classic example is the Thermographic Index (TI) concept, which uses an isotherm-based approach and explicitly incorporates the fraction of ROI area enclosed/covered by an isotherm together with temperature elevation, producing a single composite descriptor of “how hot” and “how widespread” the signal is. A recent implementation focused on TI computation and reporting in thermograms is provided in [42].

To ensure efficacy in area-based metrics, accurate segmentation of the ROI is needed in order to guarantee that all frames are aligned in the same way. Small changes to the boundaries, especially in the hands, can lead to incorrect heat stress area (HSA) or hotspot fraction assessments. Using automated ROI segmentation and realignment across sequences can greatly enhance the reliability of area-based measurements, especially when temperature changes are small or the sequences are long [43].

Reproducibility requires explicit reporting of the ROI definition using anatomical landmarks, the thresholding rule, the pixel-to-area conversion method, and whether hot regions are filtered using a minimum cluster size or morphological cleaning. Without these details, area-based burden estimates are difficult to compare across studies and are challenging to synthesize in meta-analyses.

## 3. Thermogram-Derived Biomarkers

In addition to basic summaries of the ROI, thermography-based biomarkers aim to convert precise thermal data regarding joints into quantifiable metrics, facilitating comparisons of results between individuals and across time. In the realm of rheumatic disorders, these indices are being examined as supplementary tools for diagnosis, evaluation of disease activity, and monitoring of therapy efficacy. The aim is to document inflammatory alterations in a way that minimizes observer bias and facilitates longitudinal monitoring, together with the incorporation of other clinical data [19].

Biomarkers produced from thermograms can be categorized into many conceptual classifications. Certain descriptors highlight spatial organization by representing the topology of heated areas and temperature gradients throughout the joint surface. Advanced analytical methodologies amalgamate many variables into composite indices or machine learning models, assessing them in conjunction with clinical scores, laboratory indicators, and ultrasound results. In the context of RA and associated conditions, both distribution-based and spatially informed indices are gaining recognition in the literature as promising quantitative indicators of synovitis and overall disease activity. However, extensive clinical implementation of these indicators necessitates additional validation and standardization [19,20].

### 3.1. Distribution-Based Indices

Rather than relying on a single summary value such as Tmean or Tmax, distribution-based biomarkers represent the complete temperature distribution within a joint ROI. Multiple studies indicate that diagnostic or monitoring signals in rheumatic diseases involve changes in the overall temperature histogram, including shifts in central tendency, upper-tail behavior, and dispersion, compared to healthy controls or individuals with less active disease [19].

Thermal imaging has been utilized in RA as a static technique, evaluating joint surface temperatures under steady-state settings, and as part of dynamic or functional protocols, documenting cooling and rewarming phases over time. Narrative studies suggest that these processes have been utilized in conjunction with computational methods to automate feature extraction and produce composite indices from thermograms. Distribution-based descriptors can be utilized to assess disease activity in conjunction with clinical, laboratory, and other imaging metrics [20].

A representative example of distribution-focused analysis is provided by an infrared thermography study that examined fingers in people with RA, measuring both still and moving temperatures. They created temperature distribution maps and analyzed changes during cooling and warming, including the areas under the cooling and heating curves and the difference between them. The distribution- and curve-based features demonstrated significant differences between patients with high and moderate disease activity. These results suggest that using specific temperature measurements from thermograms could be helpful for evaluating how active RA is, along with regular clinical assessments [13].

### 3.2. Spatial Pattern and Texture Descriptors

Two joint regions can have the same average temperature but have very different ways of spreading heat across the surface. Spatial pattern descriptors solve this problem by describing how thermal abnormalities are arranged within the ROI instead of just looking at global averages. Common methods describe the presence and shape of hotspots, including how many there are, how big they are, how close they are to each other, and where they are in the joint. They also use gradient-like measures to show how quickly the temperature changes between neighboring pixels. In RA, automated segmentation of hotspot regions of the hand, followed by texture-based feature extraction has shown clear differences between patients and controls. This shows that focal thermal patterns are more important than uniform warming of the whole hand region [44].

High-resolution infrared thermography (HRR) has been used to generate joint-level markers that define hotspot structures. A recent study compared a handful of methods and applied a clustering algorithm in order to identify hotspots within specific joint ROIs. Compared to power Doppler ultrasound as a reference standard, HRR measurements differentiated inflamed from non-inflamed joints and identified varying degrees of hypervascularity [45]. This demonstrates the value of HRR in assessing disease activity. The results suggest that including spatial information about hotspots can improve the effectiveness of thermographic biomarkers for detecting and monitoring synovitis.

Computer-aided diagnostic systems use spatial data to distinguish between patterns in thermal images and sometimes other types of images. One example is a machine learning framework that combines hand thermal images, RGB hand photographs, and grip-force measurements to screen women for RA. In this framework, the spatial distribution of hand temperature forms a multivariate signature that effectively differentiates patients from controls [46]. Methodological reviews of artificial intelligence (AI) in infrared medical imaging show increasing use of machine learning for feature extraction, pattern recognition, and image preprocessing. This includes removing noise, artifacts, and regions of interest, as well as enhancing fine structures. All of these are important steps for making thermography-based tests of arthropathies effective as spatial biomarkers [27].

### 3.3. Symmetry/Asymmetry Signatures

In limb thermography, it is crucial to evaluate both sides, as healthy persons generally exhibit comparable temperatures in their arms and legs under uniform conditions. Research utilizing digital infrared thermography as a benchmark has demonstrated that healthy individuals display little temperature discrepancies between limbs. Typically, absolute lateral temperature variations (|ΔT|) are below 1 °C in the majority of body areas. The temperature differentials between joints (ΔT), the temperature of the assessed region, and the temperature distribution patterns combined signify localized synovitis [37,47].

In RA, the equilibrium between systemic and local processes is evidenced by the observed patterns of symmetry and asymmetry. A study comparing persons with RA to healthy subjects revealed elevated temperatures in the hands and wrists of RA patients, exhibiting analogous thermal patterns bilaterally in the absence of active inflammation. This indicates that particular thermographic alterations may signify widespread vascular or metabolic processes [48]. In contrast, case-level analyses utilizing infrared thermography and ultrasonography revealed wrists with focal synovitis in regions approximately 2–3 °C warmer than corresponding anatomical locations on the opposite side, accompanied by an increased Doppler signal, while non-inflamed joints displayed relative symmetry [49]. The data support the use of joint-level ΔT, distinguished between an affected region and a contralateral or locally defined “reference” area, as a marker of active mono- or oligoarthritis amidst broader thermal fluctuations.

In addition to the above, RA and OA may differ in terms of the spatial logic of what may be predicted thermographically. The thermal abnormalities in RA are usually understood in terms of bilateral, multi-articular, or to some extent symmetrical inflammatory disease, primarily in hands and wrists and therefore, when simultaneous symmetrical joint inflammation is present, a purely contralateral asymmetry could underestimate activity [20]. In such conditions, multi-joint thermographic mapping and indices of distribution correlated with ultrasound definition of synovitis become more informative. In OA, and specifically in symptomatic knee OA, the thermal pattern will typically be localized, segmental, or symptom-dependent. Differences in medial, lateral, patellar, or suprapatellar temperatures reflect local mechanical stress, mild degrees of synovitis, or location of pain [41]. Thus, in RA, it is systemic/multi-articular inflammatory changes that we are primarily concerned with, and in osteoarthritis, we are mainly concerned with localized heat output from a compartment, comparisons side to side, and regional contrast with clinical findings.

Thermography becomes harder to interpret in RA-OA comorbidity, where localized heat of compartment/degenerative disease is overlaid upon the general heat of inflammation. Perhaps in the case of a simultaneous hand/multi-joint increase of inflammation temperature corresponding with RA, there is not enough discrepancy between the region temperature and a localized hotspot produced by a compartmental hotspot of OA, whereas localized heat corresponding with an OA compartmental signal might be mistakenly taken for localized inflammation, which does not correspond to its clinical distribution. For this reason, RA or OA cannot be definitively attributed by the presence of a mixed thermal signal to one specific condition with concurrent illness, and thermographic interpretation needs to be supplemented with the interpretation of regional thermal pattern, symptoms, and structural findings, as well as the presence of ultrasound evidence of synovitis/Power Doppler.

**Figure 2:**
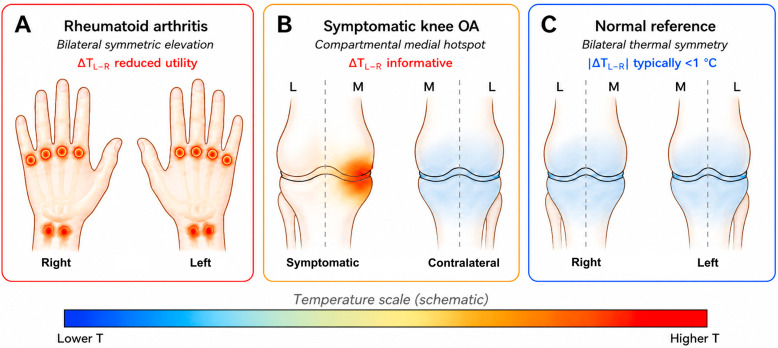
Schematic illustration of representative thermographic patterns in arthropathy assessment. (**A**) Bilateral symmetric elevation at the metacarpophalangeal and wrist regions in rheumatoid arthritis RA, reflecting primary synovitis sites as defined in thermographic scoring frameworks; the symmetric distribution reduces the diagnostic utility of simple contralateral temperature comparison (ΔT_L–R_). (**B**) Compartmental medial hotspot with relative lateral cooling in symptomatic knee osteoarthritis; ΔT_L–R_ is clinically informative and amenable to contralateral normalization. (**C**) Bilateral thermal symmetry in a normal reference limb, with |ΔT_L–R_| typically below 1 °C, providing the normative baseline for asymmetry index calibration. L = lateral; M = medial.

Research beyond rheumatology has identified additional evidence that thermographic asymmetries are significant for overall health. A modest investigation of individuals with chronic stroke and hemiplegia revealed distinct temperature disparities between the affected and unaffected lower limbs. Following a single session of strength training, these discrepancies diminished, indicating that ΔT can reflect short-term alterations in blood flow and neuromuscular performance [50]. This study, although not directly related to rheumatology, illustrates that side-to-side thermographic asymmetry serves as a reliable marker for disease progression and treatment effectiveness. Keeping track of joint-level ΔT over time in inflammatory arthritis could help tell the difference between permanent damage, which usually looks the same on both sides and has no temperature change, and new or returning inflammation, especially when comparing one limb to the other. Figure 2 illustrates the representative thermographic patterns described in this section, contrasting bilateral symmetric elevation in RA hands with compartmental asymmetry in knee OA and bilateral symmetry in the normal reference limb.

### 3.4. Feature Selection and Dimensionality Reduction

Thermographic evaluations of arthropathies frequently produce high-dimensional information. A single thermogram of a joint can yield thousands of pixel values and diverse data, including temperature differentials, symmetry indices, textural characteristics, and cooling–warming rates. However, the sample size for model development is generally limited. In these instances, selecting appropriate features and minimizing dimensionality are essential to prevent overfitting, enhance model reliability, and facilitate comprehension. The literature on radiomics identifies these processes as vital elements of quantitative imaging workflows, in conjunction with the standardization and validation of acquisitions [51].

Feature selection finds thermographic variables that give the best information for a specific goal, such as identifying ultrasound-defined synovitis or measuring disease activity in RA. The radiomics literature identifies three primary feature selection methods. Filter strategies evaluate feature properties, such as variance or correlation. Wrapper strategies add or remove features based on predictive performance. Embedded strategies enable the learning algorithm to select features, as exemplified by penalized regression or tree-based models [52].

By reducing the original feature set and maintaining the most critical information, dimensionality reduction makes feature selection better. Principal component analysis and other methods are often used in radiomics to put similar data into smaller groups [53]. These pieces are then used as inputs for other models. In cancer radiomics, systematic reviews demonstrate the significance of meticulously selecting characteristics to prevent model overfitting and ensure compatibility with fresh data [53].

A very important stage in the evolution of thermogram-based biomarkers was the progression from discrete temperature measurements to complex thermographic scores. ThermoJIS (Thermographic Joint Inflammation Score), was developed as a machine learning-derived index of thermal activity in the hands, using ultrasound-defined synovitis as gold standard. The original study involved 595 patients recruited over 4 years in two hospitals. They included individuals with RA, psoriatic arthritis, undifferentiated arthritis, secondary arthritis of the hands, hand osteoarthritis, and controls. The machine learning model was trained and optimized on a set of 449 participants, with validation performed on a set of 146 RA patients [16]. The ThermoJIS workflow is clinically relevant because it does not depend on manual temperature sampling from predefined joint ROIs. Images were pre-processed through background removal, denoising, and enhancement. From the images, distinct thermal regions, indicating variation in heat signal, were automatically identified and processed with a k-nearest neighbors’ machine learning algorithm to determine a synovitis-specific score that correlated with ultrasound power Doppler activity [16]. The final ThermoJIS value represented an aggregated patient-level score, with higher values indicating a greater probability of active synovitis [16]. This approach attempts to capture spatial thermal patterns rather than relying only on absolute temperature, thereby reducing dependence on exact temperature calibration and manual ROI placement.

Within the validation cohort, ThermoJIS had moderate correlations with ultrasound grey-scale synovial hypertrophy and power Doppler scores. ThermoJIS detected active synovitis, defined as a grey-scale sum score > 1 and a power Doppler sum score > 0, with an AUROC of 0.78. The sensitivity at the proposed cutoff value of 3.56 was 94%, although the specificity was relatively low at 51%. The positive predictive value, negative predictive value, and F1-score were 68%, 88%, and 0.79, respectively [16]. When an indeterminate interval was introduced, excluding ThermoJIS values between 3.46 and 5.65, AUROC increased to 0.86 and specificity improved to 82%, but 43% of patients were classified as indeterminate [16]. This trade-off is important for clinical implementation: ThermoJIS may be useful as a sensitive screening or triage tool, but borderline values require either repeat thermography, clinical review, or confirmatory ultrasound rather than automatic therapeutic decisions.

ThermoDAI and ThermoDAI-CRP represent a further development from ThermoJIS towards more holistic disease assessment beyond initial inflammation detection [54]. They were designed as simple composite indices inspired by CDAI and SDAI but without formal joint counts. ThermoDAI is calculated as ThermoJIS + Patient Global Assessment, while ThermoDAI-CRP is calculated as ThermoJIS + Patient Global Assessment + CRP. In the validation cohort of 146 patients with RA, ThermoDAI and ThermoDAI-CRP showed stronger correlations with ultrasound-defined synovitis than Patient Global Assessment alone or Patient Global Assessment plus CRP [54]. Correlations with ultrasound grey-scale and power Doppler scores were 0.52 and 0.56 for ThermoDAI, and 0.58 and 0.61 for ThermoDAI-CRP. Both indices also showed strong correlations with established disease activity scores, including CDAI, SDAI and DAS28-CRP [54].

The reported disease activity thresholds were 5.3, 10.3, and 13.2 for ThermoDAI remission, low disease activity and high disease activity, and 6.4, 10.9, and 14.1 for the corresponding ThermoDAI-CRP categories [54]. Agreement with CDAI and SDAI categories was substantial, with weighted kappa coefficients of 0.73 [54]. Using remission thresholds to detect active synovitis, ThermoDAI showed sensitivity of 96% and specificity of 30%, while ThermoDAI-CRP showed sensitivity of 88% and specificity of 35% [54]. These values reinforce the potential role of these indices as sensitive remote or outpatient screening tools, especially when physical joint counts are unavailable. However, the low specificity indicates that they should not be interpreted as stand-alone diagnostic replacements for ultrasound, clinical examination, or laboratory assessment. To further clarify the development, validation design, diagnostic performance, limitations, and clinical implications of these composite thermographic indices, Table 1 summarizes the key methodological and quantitative characteristics of ThermoJIS, ThermoDAI, and ThermoDAI-CRP.

One significant limitation of composite thermographic indices is the potential for a certain degree of correlation among many potential predictors. For instance, the mean temperature, maximal temperature, variance, texture features and hotspot burden are likely to contain somewhat related information, and high performance might be due merely to the redundant feature structure or bias in protocol. In relation to the further development and validation of ThermoJIS-, ThermoDAI-, and ThermoDAI-CRP-type models, the degree of multicollinearity and the stability of each variable have to be investigated with appropriate filters (correlation filters), penalized/regularized modeling, permutation-based feature-importance stability and external validation. In addition, such indices are interpreted as indicators of the inflammatory state, rather than individual signs of synovitis.

## 4. Automated ROI Definition and Segmentation

Quantitative thermography in joint diseases relies heavily on clearly defining and consistently marking the same ROIs on joints for different patients and visits. In many current RA methods, the areas of interest (ROIs) on the joint are still drawn by hand on thermal images, and then the lowest, highest, and average temperatures are calculated from these marked areas. In a study examining thermography in the elbow for RA, trained observers marked the front, inside, outside, and back parts of each elbow on thermal images before measuring the temperature. This procedure was reproducible but required considerable effort from the operator for each patient [55]. These kinds of manual workflows take a lot of time, are prone to differences between and within observers, and are difficult to use with many joints, different time points, or studies at more than one center.

Automated and semi-automated techniques for defining and segmenting regions of interest (ROIs) have been established to mitigate these constraints, especially in hand and limb thermography. Image processing methods and geometric principles facilitate the recognition of the hand, the delineation of the fingers from the palm, and the extraction of specified anatomical regions of interest. These methods yield standardized temperature readings with minimal human involvement. The automated extraction of 44 regions of interest from thermal pictures of different anatomic regions showed significant agreement with human evaluators and decreased processing time by nearly tenfold relative to manual segmentation [56]. The use and enhancement of these automated ROI methodologies for joint-specific thermography in rheumatology may facilitate more reliable measurements, greater efficiency, and a more robust basis for subsequent feature extraction and artificial intelligence models.

### 4.1. Sources of Variability and Motivation for Automation

Quantitative thermography can yield valuable clinical insights only if we control for variations during the procedure, as the measured signal reflects the skin’s surface and may be influenced by factors unrelated to pathology. During the acquisition phase, factors such as ambient temperature, airflow, the subject’s acclimatization to the camera, their posture, and the camera’s distance and angle might influence the absolute temperatures and gradients in the joint area, even with identical cameras. These problems hinder the replication of studies and hamper comparisons across research groups [22].

Studies in the field of knee osteoarthritis thermography clearly demonstrate a wide range of differences in their methods. Recent evaluations show that different studies use different ways to get data, different camera systems, different methods to handle data, and different ways to define and analyze areas of interest. For data comparison, benchmarking, and multicenter validation to work, it is essential to use standardized methods [23].

Despite the high standardization of acquisition, the definition and tracking of ROIs introduce considerable instability during analysis. The way regions of interest (ROIs) are manually placed relies on the person doing it and can be hard to reproduce consistently by different people or at different times. In addition, when doing the same measurements multiple times or during moving activities, keeping the ROIs aligned is time-consuming and the ROIs can easily shift. Recent studies on the knee demonstrate that marker-free posture and keypoint estimates can automate the selection of regions of interest and the extraction of temperature data. This method reduces the necessity for laborious semi-automatic processes and enhances the consistency of regional measurements during repeated movements [57].

Therefore, automation is driven not only by efficiency but also by the need for accurate measurements and the ability to grow. The need for stable, reproducible region extraction becomes more important as thermography is used more often with AI-based pipelines. Segmentation and region definition serve as the “gate” through which quantitative features are learned or computed. Recent research on RA that combines thermal imaging with automated region segmentation and classification shows this trend. It shows that consistent region extraction is necessary for reliable downstream decision support in end-to-end computational workflows [58].

### 4.2. Rule-Based and Landmark-Guided ROI Extraction

An effective approach to automating clinical thermography utilizes anatomical references and key locations instead of depending exclusively on pixel-by-pixel analysis from a singular model. This method first finds keypoints, localizes anatomical regions, or employs fiducial reference points to create a reliable anatomical reference. It then uses clear geometric rules to outline important areas, including proportional boxes, balanced sections, or areas focused on joints. This system facilitates ROI placement detection, optimizes verification, exhibits diminished sensitivity to minor image flaws, and endorses full automation.

For lower-limb thermography, employing various methodologies and reference points demonstrates how to maintain consistency in the areas of interest. A new method for acquiring thermal images of the back of the leg uses a special setup to connect visual labels to thermal images, using body position and keypoints to create detailed labels for different parts of the leg (such as separating the thigh, knee, and calf), and it improves the process to keep the body parts clearly defined [59].

An approach to improve ROI boundaries involves the idea of defining the ROI as a problem of object localization and then using segmentation to achieve this. In a recent sports-medicine study, a system that uses both visual and infrared data automatically locates and separates ROIs for thermal analysis using a YOLO-based detector. This standardizes the placement of ROIs and makes region extraction repeatable, which reduces the amount of variation that comes from doing things by hand and allows for faster analysis [60].

Landmark guidance does not need to be purely algorithmic; it can also rely on fiducial anatomical reference points that deterministically constrain ROI geometry. In a recent knee thermography protocol, metal markers were placed on the legs as reference points, and the ROI was explicitly defined between the two markers for temperature extraction. This provides a clear example of a landmark-guided, rule-based ROI construction in a joint setting, where ROI placement is anchored to reproducible external landmarks to improve comparability across thermograms [61].

The latest reviews emphasize that modern thermography pipelines are increasingly combining classical processing with AI, such as object detection, feature localization, and segmentation. For this particular reason, thermal images often have blurry edges and lack structural detail. In this context, landmark-guided and rule-based ROI extraction serves as a practical “middle layer” that enhances robustness, diminishes reliance on flawless pixel segmentation, and produces ROIs that are clinically interpretable and amenable to automated analysis [27].

### 4.3. Learning-Based Segmentation and Keypoint Detection

Learning-based ROI extraction alleviates the shortcomings of manual and rule-based methods, which often fail in diverse scenarios, body postures, or anatomical variations. Owing to the ambiguous boundaries and limited structural diversity in thermographic images, models are designed to provide accurate masks that distinguish the target tissue or area from the background. U-Net-style encoder–decoder architectures, including skip connections, are frequently employed, as they integrate general context with individual specifics to provide accurate segmentation masks. These masks enable the acquisition of temperature data from specified areas or define critical regions around joints using known protocols after initial processing [62].

U-Net and its derivatives are still widely used in biomedical image segmentation because they strike a good balance between accuracy and robustness across different types of datasets. A recent thorough review shows that U-Net-based architectures remain the most common reference frameworks in medical segmentation. It also explains how modern versions are made to work better with limited or noisy data [63]. This feature is especially important for thermographic applications because the datasets are usually smaller than those in radiology, and the performance can change when the domain changes, for example, when the camera, background, or acquisition protocol changes.

In rheumatic disease thermography, automated workflows typically standardize region extraction prior to drawing any conclusions. For example, thermograms of hands with RA have been processed in a special way to separate the hot areas from the background using grouping techniques, followed by identifying important features and using machine-learning classifiers [64]. The evidence shows that defining ROIs using an algorithm can make the analysis less sensitive to changes in framing and provide more consistent inputs for classification. Along with single-cohort development, external validation studies of thermographic indices derived from machine learning, such as composite joint inflammation scores calculated from hand thermography, demonstrate that it is possible to test algorithmic outputs outside of the development environment. This strengthens the case for reliable and generalizable automated thermography workflows [15].

Keypoint or landmark-based detection can work together with segmentation by using a few specific anatomical reference points to create a reliable coordinate system. This approach can also continue to benefit from its acquired resilience to pose variations.

Thermography pipelines based on learning should be evaluated as measurement systems rather than solely as computer vision models. The Dice similarity coefficient and Intersection-over-Union (IoU) are two common overlap metrics used to measure the quality of segmentation. On a scale from 0 (no overlap) to 1 (full overlap), these metrics show how much the predicted mask and a reference mask overlap. However, it is essential for clinical use to understand how mistakes in segmentation or the region of interest (ROI) affect thermographic indicators and whether the results meet important clinical needs. Evidence at the knee level comparing thermographic temperatures with ultrasound-detected inflammation in RA clearly illustrates the significant correlations between assessed regional temperatures, supporting the idea that automated ROI/segmentation can produce consistent and reproducible measurements [65].

To clarify the practical differences between ROI strategies, Table 2 compares manual ROI selection, rule-based ROI construction, landmark-guided extraction, and learning-based segmentation in terms of reproducibility, feasibility, expertise requirements, and robustness.

Overall, manual and rule-based methods appear more suitable in the restricted environment of small studies, whereas learning-based or landmark-based methods might be more appropriate for longitudinal, multi-center, or AI-driven workflows, as long as explicit risk-monitoring of method failures, errors, and sensitivity to domain shifts is implemented.

### 4.4. Quality Control and Failure Detection

Even with carefully engineered ROI definitions, joint thermography remains inherently vulnerable to technical artifacts and protocol deviations. Recent scoping work on medical thermography shows that published studies still vary widely in camera specifications, environmental control, and patient preparation, and that these technical details are often incompletely reported, making replication and cross-study comparison difficult. The same review emphasizes that thermography should be treated as a full measurement process, with technical and environmental settings explicitly catalogued and linked to diagnostic performance and reference data [24]. Quality control within arthropathy studies must therefore be established in advance and precede all image analysis and inferences from AI. This check would confirm the correct imaging range and stability in camera calibration, camera location, environmental variables, patient adaptation, identification of the joint for imaging, absence of obstructions (occlusions/reflective surfaces) and location of the region of interest (ROIs). According to the specified criteria, each picture has to be classified as ‘technical validity’, ‘excluded,’ or ‘cautious analysis required’ (in cases of low confidence and uncertainty of AI results).

At the acquisition level, scoping reviews list a wide range of technical and environmental factors used in medical thermography studies. These include the type of camera, the resolution of the detector, the spectral range, the calibration procedures, the ambient temperature and humidity, the length of time it takes for the patient to become accustomed to the procedure, and the patient’s position. This variability shows how flexible the technique is and how important it is to standardize it [24]. Protocol rules must include these factors for joint imaging. These include the acceptable ranges of ambient and skin temperature, the minimum time needed to get accustomed to the new environment, the set distance and angle of the camera to the joint, and clear instructions on what to wear, what jewelry to wear, and what topical applications to use.

Quality control ensures the thermogram image is clear enough to view the joint properly. This means that the target joint and surrounding area must be visible, the frame should not cut off any part, and bandages, dressings, or reflective items should not block the view. In AI-enhanced thermography pipelines, reviews of infrared imaging and sensing outline steps for data cleaning and quality assurance. These steps include removing images that are blurry, blocked, or incorrectly captured before model training or inference, as well as standardizing the selection of regions of interest [27]. For arthropathies, similar filters can automatically check basic image qualities, such as whether the area is visible, if the image is too bright, or if there is motion blur. Simple rules can also be used for the joint area of interest, such as its size, location in the body, and whether both sides are symmetric.

To ensure quality in learning-based workflows, it is essential to regularly check both thermographic indices and model outputs. A new study used machine learning to construct thermographic indices from images of hand joints that had RA. After that, the resulting indices were validated with a separate group of patients. The researchers set clear standards for the patients and photos they examined, and followed a set methodology to ensure that only the right thermograms were examined [15]. Applying this method to new data establishes the reliability of thermography-based measurements, particularly for certain joints. It makes it easier to identify patients, the imaging process, and the standards for selecting images. In practice, there needs to be clear eligibility criteria for both patients and photos that correspond to each thermographic index or artificial intelligence score. Quality control documentation must list the frames or visits that were excluded and provide explanations for their exclusion. For example, they may have been omitted due to protocol violations, incomplete images, or serious photo issues.

Finally, modern AI governance emphasizes that quality control and failure detection are inseparable from transparent reporting. Comprehensive reporting guidelines for AI models in healthcare now recommend that studies describe the entire pipeline, from data preparation and cleaning to model training, evaluation, and clinical implementation, including how outliers, missing data and technical errors were identified and handled [66].Applying such guidance to joint thermography means that authors should not only present model performance and thermographic biomarkers, but also document how many images and patients were discarded at each QC step, what criteria were used to flag failures, and how uncertain or low-confidence outputs were treated (for example, “no-decision” cases sent to human review). When automatic ROI and segmentation are combined with explicit, well-reported QC procedures, joint thermography moves closer to a robust measurement system: one in which technical instability is actively monitored, failure modes are recognized rather than hidden, and the resulting temperature-based indices can be interpreted as reliable reflections of joint physiology.

### 4.5. Impact of Automated ROI Definition on Reproducibility and Measurement Error

In thermography, a good choice of the Region of Interest (ROI) can contribute to a cleaner and more reliable picture, more reliable measurements in repeated test sessions, and the minimum detectable temperature changes compared to background noise levels. Standardization in the available studies evaluating thermography reliability proved that appropriate control over ROI localization can yield highly reproducible data as seen by intraclass correlation coefficients (ICC) ranging from 0.82 to 1.00 in various upper-extremity ROIs [67], and in excess of 0.94 for the mean skin temperature values between novice and experienced operators [68]. However, this evidence is supplemented by findings demonstrating that the manual method of ROI delimitation still generates intraobserver error for minimum temperature and for temperature-range measures. This means that reliability depends on operator competence and training in addition to replicability of the ROI shape, pixel inclusion and exclusion among different test conditions, subjects, joints, and observers.

When the ROI geometry is algorithmically determined, residual measurement error can be quantified and minimized. In a study utilizing infrared thermography to assess athletes’ triceps surae, skin temperature was measured in 1 cm^2^ regions twice on the same day and again 48 h later, using the same device and protocol [69]. The intraclass correlation coefficients (ICCs) for both intra- and inter-session measurements ranged from 0.968 to 0.977. The standard errors of measurement ranged from 0.19 to 0.23 °C, and the smallest detectable changes ranged from 0.52 to 0.64 °C. These findings establish that, when ROI position and size are consistently replicated, small variations in local temperature are meaningful.

In RA, composite thermographic indices show that algorithm-driven analysis can measure disease activity over time. The Thermographic Disease Activity Index (ThermoDAI) and ThermoDAI-CRP use machine learning to analyze heat images of the hands, and ThermoDAI-CRP also includes C-reactive protein. In 146 patients, these indices showed a fair to strong relationship with ultrasound scores for joint inflammation (ρ ≈ 0.52–0.61) and a strong relationship with other disease activity measures (ρ > 0.81). They also had high sensitivity for detecting active joint inflammation [54]. The thermographic component comes from standardized analysis of the same anatomical areas in each image. This removes differences in joint counting and region-of-interest placement. ROI placement is no longer needed, making it easier to compare data over time and reducing the chance of measurement errors at the index level.

Data from rheumatoid knees support a correlation between standardized thermal characteristics and objective measures of inflammation. A recent study gathered heat patterns from certain knee areas in patients with RA and compared them to inflammation detected by ultrasound. The thermographic data showed a moderate link with ultrasound results, suggesting that steady temperature patterns in certain areas could be positive signs of joint inflammation [65]. Although the study used manual ROI placement, its methodology highlights the importance of spatially uniform sampling. Automated segmentation of the area around the knee could make it easier to obtain consistent results over time, improve agreement on temperature readings, and lower the smallest change that can be detected for joint inflammation.

## 5. Machine Learning and Deep Learning for Prediction and Phenotyping

ML and deep learning (DL) further enhance cutaneous thermography by turning raw temperature data into actionable forecasts and identifying disorders. Models can do more than just examine temperature data from specific areas. Models can analyze temperature trends, limb differences, and specific features to provide results that support important medical tasks, such as detecting inflammation, estimating disease activity, and determining whether additional imaging is needed.

The clinical usefulness of these models depends on the choice of target endpoints and the accuracy of the data used to develop them. When evaluating joint diseases, comparing predictions to imaging reference standards yields the most valuable results. To avoid confusion and make the results more useful, it is important to have consistent data collection and clear quality control (QC). Feature-based machine learning, which uses specific characteristics of data to make predictions, and image-based deep learning, which employs neural networks to analyze images, are two different but useful ways to learn. Using them together works better when we can explain their results and understand any doubts we have about them. This is because it makes sure we can trust the results.

### 5.1. Clinical Tasks and Target Endpoints

To use ML and DL with skin temperature imaging in joint diseases, it is essential to clearly define important tasks and goals. Thermography records skin temperature patterns that suggest changes in blood flow and metabolism, not direct inflammation of the synovial membrane. Therefore, we must clearly tie the model’s objectives to specific clinical questions and results. Clinical prediction studies that ML and DL should have their own reporting standards to ensure that these models are clear, reproducible, and easy to understand [70].

Common tasks include sorting joints or patients into inflamed or non-inflamed groups, using regression to measure inflammation levels continuously, and using thermography to identify which patients need further imaging or closer monitoring. The chosen reference standard significantly affects the perceived effectiveness of these activities and their consequences in a therapeutic setting. However, these goals do not directly show the amount of inflammation that is occurring.

When the goal is to find or measure active inflammation, imaging-defined reference standards are the best choice. The importance of ultrasound in RA is that grey-scale synovial hypertrophy reflects structural synovial thickening, while Power Doppler activity indicates inflammatory vascularity. These two aspects of ultrasound provide a more direct image of synovitis than that given by thermography, which indicates distribution of temperature across skin by means of heat, which is subject to influences of perfusion, metabolism and heat transfer, not of the synovial tissue. Ultrasound evaluation of synovitis, including power Doppler activity, is a common way to objectively measure inflammatory activity. It also makes a strong endpoint for training and validating machine learning and deep learning models based on thermography. The construct validity of patient-level inflammation outcomes obtained from EULAR-OMERACT joint-level ultrasound scoring has been established in RA cohorts, affirming their utility as significant targets for predictive modeling [71]. Similar ultrasound-based synovitis scoring systems have been assessed in various arthropathy contexts, including hand osteoarthritis, thereby affirming the broader relevance of imaging-defined inflammation endpoints across multiple disease entities [72]. Thermography should, therefore, be seen, at present, not as a stand-alone surrogate marker to substitute US-defined synovitis but as an aid in screening and monitoring to allow further investigation of joints or patients with elevated temperature. As such, thermography could be complementary to gray scale and power Doppler US in that it will be able to deliver quick, non-contact, repeatable functional signal in the course of triage, follow-up, and target selection for confirmation of imaging. However, one needs to weigh this translation interpretation with possible false-negative cases as well.

A balanced translation of evidence into clinical practice requires addressing possible false-negative circumstances. Since thermography only measures surface temperature and does not directly reflect synovial joint status, an abnormal thermal picture will not always be present in disorders that primarily manifest with central pain sensitization or central pain mechanisms, or where an inflammatory process is at its earliest, subclinical stages (e.g., very early seronegative spondyloarthritis and fibromyalgia [73,74]). In such cases, a normal thermal picture will be obtained in the presence of clinical pain. Thus, the normal thermogram should not be interpreted as ruling out a significant, clinically interesting condition and needs to be integrated with the clinical examination, appropriate investigation such as imaging (ultrasound, MRI) and blood markers.

Besides choosing endpoints, studies need to clearly state what they are predicting, such as at the joint level or patient level, and explain how it will be used in healthcare, such as for screening, triage, monitoring, or predicting treatment outcomes. Measures implemented to ensure data quality should also be detailed. Standardized methods for collecting data and clear quality control (QC) steps are important because thermographic signals can be affected by the environment and changes in the body. Structured assessments of bias and relevance, designed for AI-based prediction models, are increasingly being used to ensure thorough evaluation and application in clinical settings [75].

### 5.2. Feature-Based Machine Learning Models

Classical classifiers or regressors utilize feature-based ML pipelines to transform thermograms into a succinct set of manually crafted descriptors (features). In rheumatology thermography, these features are usually calculated after the area of interest (such as joint-centered patches or hotspots) has been defined. They include temperature statistics (such as the minimum, maximum, average, and standard deviation), differences between the left and right sides, and descriptions of how heat is distributed. The mapping from pixels to features is clear, so it is easier to audit and understand these pipelines than end-to-end deep learning [19].

A typical procedure includes (i) dividing up the important areas into smaller chunks (for example, using clustering to discover hotspots), (ii) extracting features, and (iii) training the system with models like SVM and ensemble methods. For instance, hand-thermography work has employed clustering to locate hot regions and looked at several feature extractors and classifiers, like SVM and boosting/bagging. These classifiers did a superb job of sorting data when they were cross-validated [64]. Thermographic indices that show joint inflammation and disease activity for remote assessment in RA have been produced using feature-based modeling [54]. These indices correlate with clinical activity metrics. Recent research suggests that it may be possible to do longitudinal external validation of thermographic indicators based on ML. This immediately meets the need to verify robustness over time and across diverse patient groups [15].

Beyond the SVM and the pipeline approaches using ensembles, other feature-based thermographic models can also be implemented, such as with logistic regression, decision trees, random forests, k-nearest neighbors, Bayesian classifier, or through methods guided by feature selection. However, the clinical feasibility of these algorithms varies. Logistic and penalized regression models could be useful if interpretability and calibration is important, while tree-based and random forest models could be used to exploit non-linear relationships between statistics, asymmetry indices and texture features. K-nearest neighbor may be useful to probe pattern recognition, but it is very dependent on normalization and the structure of cohorts. For poor thermographic signals and conditions, the uncertainty is given explicitly by Bayesian models. Therefore, “the most clinically relevant feature-based approach is not determined solely by the mathematical complexity of the algorithm”, but clinical relevance will also be based upon the reliability of the features, interpretation and calibration, individual patient-based determination, and clinical relevance with reference data [52,64].

For feature-based models to work well in clinical settings, it is crucial to carefully manage differences that can occur before analysis, such as the environment, how patients are prepared, and the distance and angle of these factors. These factors can systematically alter temperature-derived features and reduce generalizability. Systematic reviews have found major differences in thermography methods and show that it is important to have consistent procedures and external checks when examining the reported results [19].

### 5.3. Deep Learning for Thermographic Analysis in Arthropathies

DL methods use models that automatically extract important features from thermograms, rather than relying on manually created thermographic descriptors. However, effective DL processes in joint diseases still need careful preparation, such as adjusting temperatures, standardizing image quality, and, most importantly, clearly defining the ROI. Without this, background and acquisition-related artifacts can overwhelm the learned signal. Recent approaches to studying RA show that it is important to preprocess data by identifying specific regions in thermograms, such as separating joints or identifying hotspot regions, before training and comparing deep learning models [58].

DL is often used in recent studies of RA to distinguish between RA cases and control groups. The dataset and training methods used to create these models, such as data augmentation, partitioning, and validation, have a bigger impact on how well they work than the model architecture itself. A recent study published in *Scientific Reports* developed a custom convolutional neural network (CNN) using hand thermograms, assessed its performance relative to pre-trained models, and employed the CNN to extract features for traditional machine learning classifiers. This method addresses the problem of small sample sizes by using deep learning to carefully extract features, rather than viewing the process as a black box [76].

**Figure 3:**
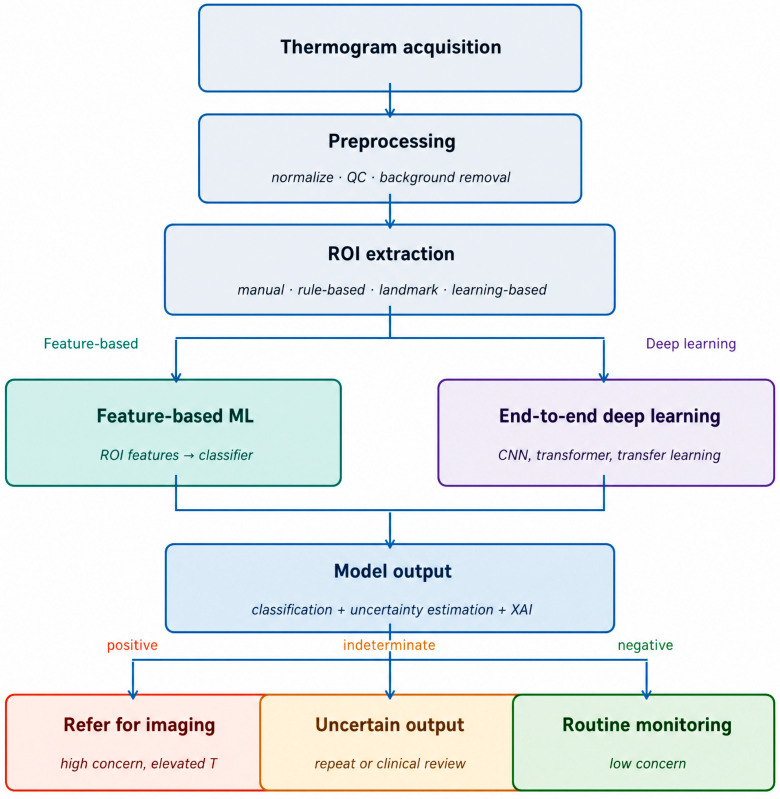
Schematic overview of the computational pipeline for thermographic arthropathy assessment using machine learning (ML) and deep learning (DL).

More recent deep-learning approaches to thermal arthropathy classification have evolved beyond simple CNNs to distinguish between types or grades. For low-data environments, a transfer-learning model, a pre-trained-features model, or standard models pre-trained on other types of images may have higher applicability, but domain-matching issues may occur among natural, radiological and thermographic images [76]. CNN-Transformer-like architectures might benefit from considering wider areas spatially, providing increased potential coverage of localized heat patterns of the hand or even over several joints. However, these architectures tend to require more training data, increase the risk of overfitting, and may require independent verification [58]. Lightweight models could be highly promising for point-of-care devices, as they have low computational cost and can be run on a mobile device. However, there may need to be a trade-off between efficiency and accuracy, and it would be essential to calibrate. The embedded deep learning prototypes, where images are directly parsed on-device with an embedded inference engine, look tempting for initial tests and even for use as on-device diagnostics if proper control over image quality, error diagnosis, and uncertainty estimation is available before clinical deployment [12,27]. Figure 3 shows the generalized computational pipeline from thermogram acquisition through ROI extraction, ML and DL analysis, and model output to clinical decision.

Another area of development is the “deployment-aware” usage of DL with thermography, where the algorithm is built into a portable process that is meant for early screening or helping with first decisions. A recent prototype combines thermal image acquisition with a built-in deep learning inference pipeline for early screening of arthritis and arthrosis. It also reports on clinical evaluation under controlled acquisition settings [12].

Recent reviews of medical thermography that focus on AI show that deep learning (DL) is being used at various steps in the thermal imaging process, including improving quality, handling issues, detecting and locating problems, and supporting decisions. However, these reviews also point out ongoing challenges are particularly important for joint diseases: small sizes of datasets, differences in how images are taken with various devices and settings, and the need for outside testing if the models are meant to work in different situations [27]. Table 3 provides a comparative overview of ML, DL, and feature-based computational approaches discussed in this section, including sample sizes, validation designs, and key reported metrics.

### 5.4. Multimodal Fusion with Clinical and Imaging Data

Multimodal artificial intelligence frameworks combine images with clinical information, such as symptoms, exam results, test outcomes, and treatment history, to improve the accuracy and usefulness of diagnosis. Nevertheless, these systems demonstrate other problems, such as inconsistent data quality, missing data, and an uneven distribution of diverse data types. Failing to address these problems during study design and validation may lead to inaccurate conclusions [77].

In RA, ultrasound-based multimodal prediction shows how important it is to use more than one source of information. Hand ultrasound characteristics, combined with machine learning, have been utilized to predict the progression of RA during follow-up, demonstrating the conversion of imaging-derived inflammatory data into individualized risk assessments. This approach could encourage the use of thermography because the patterns it reveals provide additional, easy-to-access information that improves current imaging and clinical predictions, rather than just repeating them [78].

A second representative approach explicitly integrates clinical variables with structured ultrasound scoring. In this approach, models use initial clinical assessments and ultrasound scores from multiple joints to forecast the development of RA in patients with undifferentiated arthritis. Importantly, this work also shows that there is a growing expectation that multimodal models should provide interpretable variable attribution (for example, SHAP-style analysis) to help determine whether predictions are based on clinically plausible imaging and clinical factors. This supports both trust and error analysis [79].

In multimodal fusion, data leakage, cohort bias, or a lack of transparency in preprocessing can all make performance metrics appear better than they actually are. Therefore, it is essential to keep detailed records. Imaging-AI reporting checklist authors must state the AI system’s purpose, data source, inclusion criteria, and evaluation and validation methods. These requirements are very important when thermography is used in conjunction with ultrasound, clinical scores, and laboratory tests [29].

### 5.5. Explainability and Uncertainty Estimation for Clinical Trust

Clinical settings require AI systems that use cutaneous thermography to be understandable. Thermographic patterns can be altered by non-disease factors, such as the conditions under which they were taken, the room temperature, and body variability. To help people understand and trust AI systems that use cutaneous thermography in healthcare, we need explicable AI (XAI) methods to show which areas of the images, temperature patterns, or features have the biggest impact on the predictions. In medical imaging, explainability primarily aims to enhance transparency and facilitate error analysis, rather than to establish causal reasoning. Recent reviews show that post-hoc explanation methods, especially saliency-based visualizations, are quite sensitive to changes in the model and how it is set up. As a result, thermography studies need to clearly outline the explanation method they are using, state what they aim to achieve (such as troubleshooting, ensuring quality, or helping doctors), and not assume that easy-to-understand explanations mean that they are biologically accurate [80].

It is important to assess uncertainty accurately to ensure the safe use of artificial intelligence applications. Sometimes, AI models are too confident when they encounter new data, even if their predictions and explanations appear reasonable. Even small changes in hardware, imaging protocols, environmental conditions, or patient populations can change the data distribution, which is a major challenge for thermography. Common ways to measure uncertainty include ensemble modeling, Bayesian methods, and test-time augmentation [81].

Clear reporting standards are essential to ensure that explainability and uncertainty metrics truly help in understanding clinical results, rather than being used later as excuses due to selective reporting and inconsistent use. Recent reporting requirements for AI-driven diagnostic accuracy research require authors to detail data provenance, dataset segmentation, the definition of the AI “index test,” evaluation methodology, and sources of bias and application issues. In thermography applications for arthropathies, it is essential that explainability methods, uncertainty management, and decision thresholds are clearly defined to allow readers to evaluate the proposed system’s alignment with practical clinical workflows and acceptable risk parameters [82].

Lastly, for thermographic AI systems to be trusted in clinical settings, they need to be built and tested in a way that takes the whole lifecycle into account, not just the model. Every step of the process, from designing and testing the system to getting it approved and using it, should include understanding how it works, ensuring it is strong, and dealing with uncertainties. This includes checking for changes in performance after the system is in use. In cutaneous thermography, the quality of the signal is very sensitive to how it is acquired, making it especially important to maintain a lifecycle view that emphasizes the need to continuously monitor performance rather than checking it just once [83].

## 6. Validation, Reporting, and Reproducibility Considerations

Validation serves the purpose of confirming the generalizability of thermography-based machine learning models. Machine learning models derived from thermography images can be said to be generalizable when they achieve reasonable prediction performance outside of the training dataset and protocol. With this aim, reproducibility is presented here at the level of model validation and comprises the concepts of internal and external validation, domain shift, reporting level, and clinical decision thresholds.

### 6.1. Internal Validation and Risk of Overfitting

The initial stage in assessing whether a ML or DL model trained on skin temperature data is identifying a pattern that remains valid when evaluated with novel data. In predictive modeling, internal validation is mostly employed to mitigate the “optimism” that occurs during the selection and development of a model. This procedure is generally accomplished by resampling techniques, such as k-fold cross-validation or bootstrapping. The dependability of internal estimates depends on the congruence of the evaluation framework with the real clinical unit of inference. In thermography datasets, it is customary to gather numerous thermograms or several joint regions of interest for each participant. Consequently, to prevent inadvertently evaluating the model on data linked with training instances, the assessment must guarantee patient-level segregation [84].

When data leakage happens, information that should not be available during training accidentally affects the model. This situation can happen if data from the same patient is split across different folds, if normalization or feature extraction is done on the whole dataset before splitting, or if the model is tuned repeatedly based on test results. These issues can make a model appear very accurate during testing, but the model may not perform well when tested again or on new data. Data leakage is a major reason why model performance drops when tested outside the original experiment. In fact, many studies in machine learning have found that data leakage often leads to results that cannot be reproduced [85].

Enhanced model flexibility with regard to data volumes heightens the likelihood of overfitting. This challenge is significant in thermography-based arthropathy research, as datasets are often limited and data collection procedures vary. This variant may lead deep learning models to prioritize details from testing procedures or devices over significant temperature trends associated with those procedures or devices. Systematic assessments of deep learning research in healthcare indicate that robust assertions regarding model performance frequently accompany inadequate reporting and substandard study methods. Consequently, confidence in the reported results wanes, since they may exclusively reflect model performance on a specific dataset rather than its general applicability [86].

For these reasons, good internal practice is not simply “running cross-validation” but to structure evaluation so that the entire analytic pipeline is insulated from test information. All data preparation, decisions about regions of interest based on data, choosing features, and adjusting settings should only occur within the training groups, and results should show a range of performance across different samples instead of just one best result. At the same time, thorough reviews in medical imaging highlight significant differences in how studies are designed and reported, which means that we should be careful when interpreting results that have been internally validated. While internal validation can show that something is possible, it should be seen as just the first step that needs to be backed up by more solid evidence in later stages [87].

### 6.2. External Validation and Cross-Site Generalizability

External validation is the key step in determining whether a model that performs well internally can be expected to behave reliably in new data that were not used in any part of its development. For machine learning and deep learning in healthcare, the procedure typically involves testing the final model (and the methods used to prepare the data) on a different group of patients and reporting not only how well it distinguishes between different results but also how accurately it predicts chances and measures its performance. A common issue is that many external validation datasets are too small, which makes performance estimates unreliable and hides important issues; therefore, external validation should be conducted with a large enough sample size to ensure accurate estimates, rather than simply following general guidelines [88].

Cross-site generalizability is often weaker than internal validation, especially in imaging-based AI. Changes in population case mix, acquisition protocols, and equipment introduce a distribution shift unfamiliar to the model. Research in radiologic deep learning shows that applying a model to new data often reduces performance, even when the task is unchanged. The result indicates that a high area under the receiver operating characteristic (ROC) curve (AUC) does not guarantee that the model will perform well in different situations. This finding highlights the importance of viewing external validation as a stress test of real-world robustness rather than mere formality [89].

Although the clinical evidence for thermography in arthropathies has expanded, the field remains dominated by exploratory, single-center, cross-sectional or internally validated studies. Several studies report encouraging diagnostic performance, including AUROC, sensitivity, specificity, classification effectiveness, or correlation with ultrasound findings; however, these metrics should be interpreted cautiously because many datasets remain small, disease spectra are narrow, and acquisition protocols are heterogeneous. In particular, proof-of-concept ML/DL studies that distinguish RA from healthy controls may overestimate real-world performance because they often use relatively clean case-control designs rather than clinically mixed populations that include osteoarthritis, fibromyalgia-like pain, treated RA with residual symptoms, early sero-negative disease, Raynaud-type vascular changes, or overlapping inflammatory and degenerative pathology [19,45,64,76]. Thus, the current clinical evidence supports thermography mainly as an encouraging adjunctive screening, monitoring, and triage modality, rather than as an independently validated diagnostic replacement for ultrasound, MRI, or clinical examination.

External validation is especially challenging in thermography because the test is not only algorithm-dependent but also acquisition-dependent. Differences in camera model, detector precision, thermal sensitivity, calibration method, acquisition distance, ambient conditions, patient acclimatization, joint positioning, ROI definition, and preprocessing can all change the measured temperature distribution before the AI model is applied [22,24,90]. Therefore, external validation should evaluate the locked model together with the complete imaging and preprocessing pipeline, rather than only the classifier. The external validation of ThermoJIS/ThermoDAI-type indices represents an important step because it tests previously developed thermographic indices prospectively across multiple hospitals and during longitudinal follow-ups [15]. Nevertheless, broader validation continues to be necessary across independent centers, different camera systems, different operators, remote or home-based acquisition conditions, and wider disease phenotypes.

Future clinical validation studies should therefore move beyond reporting global accuracy alone. They should report patient-level splits, site-stratified and device-stratified performance, calibration, PPV, NPV, indeterminate-output rate, image rejection rate, subgroup performance, and agreement with reference standards such as ultrasound Power Doppler, grey-scale synovitis, MRI inflammation scores, and longitudinal clinical outcomes [82,88,89,91]. They should also evaluate clinical utility, for example, whether thermography reduces unnecessary ultrasound referrals, improves early triage, detects subclinical inflammation, supports remote monitoring, or changes treatment decisions safely. Without such evidence, high internal accuracy continues to be insufficient for clinical translation; with it, thermography could be positioned more realistically as a workflow-integrated functional imaging adjunct.

Finally, external validation is not just a statistical exercise; it is also an operational one. Models need to be tested in the area where they will be used to determine if they are performing worse in that specific location and to ensure they can be safely added to workflows. Recent guidance in radiology AI emphasizes the imperative to create systems that facilitate structured, evidence-based local evaluations (including regulations and oversight) before the widespread deployment of algorithms. A comparable methodology of standardized local validation prior to implementation facilitates the reconciliation of multi-center research findings with dependable clinical application [15,91].

### 6.3. Domain Shift and Protocol Heterogeneity

Domain shift refers to changes in the data distribution between the stages of model development and deployment. This problem is a major factor contributing to the occasional underperformance of medical AI. There are two main types of shifts: covariate shift, which happens when changes in the equipment or methods used to take pictures change how they appear, and concept shift, which happens when changes in how images are labeled or how different readers interpret them break the link between the image and its label. Recent research aimed at improving the classification of medical images has concentrated on these specific improvements. They illustrate that tackling domain shift requires both novel strategies, such as domain generalization methodology, and improved evaluation techniques that transcend a single center or data type [92].

A concrete and repeatedly observed form of covariate shift is scanner/protocol domain shift, where the same nominal task is performed on images acquired on different devices or under different acquisition protocols. A broad experimental study across common radiological modalities shows that models trained on data from one scanner typically perform worse on data from another scanner, and it quantifies this degradation across datasets and modalities, demonstrating that scanner differences alone can be sufficient to break apparent robustness. The keypoint for thermography research is simple: if thermal data has certain hardware or protocol characteristics, a model might learn these characteristics instead of the patterns related to diseases, leading to poor performance when the data collection conditions change. Recent research has focused on improving the classification of medical images, concentrating on these specific improvements [93].

In cutaneous thermography for rheumatic illnesses, protocol heterogeneity is not just a theoretical concern but a persistent limitation of the database. A recent systematic review states that infrared thermography is used in many contexts and environments. Variability includes factors such as patient preparation, environment management, camera specifications, region of interest, and full report generation. All of these factors combined create “hidden domains” within and between datasets, making it impossible to distinguish true physiological signals from changes that occur during acquisition [19].

To structure the impact of acquisition heterogeneity, Table 4 summarizes how common acquisition-related factors may affect diagnostic thresholds, AI model generalizability, and cross-device reproducibility in thermography-based arthropathy assessment.

Overall, differences in acquisition could result in an offset in absolute temperature values, discrepancies in the derived values for ROIs, and shifts in image properties because of varying equipment. Therefore, the generally accepted thresholds or AI models produced on the same protocol, center, and/or instrument environment may not be directly used without retraining, and without revalidating externally and/or testing inter-device reproducibility.

These concerns seem to be more relevant to existing thermographic indices (e.g., ThermoJIS, ThermoDAI, and ThermoDAI-CRP). It should be noted that models calibrated to a particular set of research-grade instruments, temperature processing pipelines, and ROI settings are not necessarily camera-agnostic. Once implemented in inexpensive portable devices or embedded screening systems, variations in sensitivity, spatial resolution, image noise, optics and software calibration may result in vastly different absolute temperature measurements and learned spatial characteristics. Clinical interpretation should only follow technical calibration or re-calibration of the thresholds and on images obtained with the deployed system.

In order to address concerns regarding methodologies and the requirement for a quantitative comparison of the reports, an overview of representative studies that applied thermography for the assessment of arthropathies, grouped by ROI and analytical methodology, with the outcome quantification and limitations, is shown in Table 5. This is demonstrably evident in terms of variable performance for thermography, dependent on the protocol of recording, the type of camera used, how ROI are drawn, the definition of the gold standard, the testing approach, and the phenotype of the disease being evaluated.

The accuracy and repeatability of temperature estimates in infrared thermography depend on the characteristics of the device. Experimental comparisons of thermal cameras with different spatial resolutions and tests of operator repeatability show that camera resolution has a significant effect on how well devices agree with each other and how consistent measurements are. Controlled conditions reduce variability caused by the operator, while systems with lower resolution show less agreement and are more likely to miss temperature patterns. These results are crucial for the development and deployment of predictive models. Research employing thermography and artificial intelligence must explicitly specify the device type and its resolution. These details significantly influence the results, especially when high-quality sensor models are used with cheaper devices in screening or point-of-care situations [90].

### 6.4. Clinical Utility and Decision Thresholds

AI studies using thermography in arthropathies are often presented as tools for diagnosis or triage. To assess the reliability and utility of published results, studies must be reported clearly. The STARD-AI guideline builds on the classical STARD framework by adding AI-specific items, such as dataset practices, the definition of the AI index test, evaluation procedures, and thoughts on algorithmic bias and fairness [82].

In addition to assessing the completeness of reporting, it is crucial to specifically evaluate the risk of bias and applicability. PROBAST + AI provides an enhanced instrument for prediction-oriented models, including AI and ML methodologies, featuring signaling inquiries for participants, data sources, predictors, outcomes, and analysis. It facilitates both model construction and assessment. This guarantees consistent evaluation of studies and enhances the quality of evidence synthesis [75].

Even when reporting checklists exist, real-world adherence is often incomplete. A recent review of studies on the CLAIM checklist in medical imaging AI showed that many important details were often missing, with about one-third of the checklist items left out on average, and several items were frequently unreported. This issue is particularly important for thermography ML/DL papers: if the details about how data was collected, prepared, divided, and tested are not reported consistently, it is challenging to determine if the results can be applied in different situations or repeated with other equipment [94].

A mere narrative description does not ensure reproducibility. It necessitates the documentation and, where possible, dissemination of essential elements required for autonomous reimplementation, encompassing data management, preprocessing, training parameters, and evaluation code. A recent study utilizing the Delphi approach, a structured communication technique used to reach a consensus among experts, discovered 26 characteristics that promote discussions around deep learning in medical imaging. The objective was to elucidate the processes of model creation and evaluation, thereby facilitating replication by others. It is essential to record results in a way that makes it easy for others to replicate the findings for thermography AI, due to the many different methods and device effects involved. The objective is to prevent performance outcomes that diverge from verifiable standards established by others [95].

For real-world deployment, thermography-AI systems should be evaluated according to a predefined intended use, such as screening, longitudinal monitoring, triage for ultrasound, or treatment follow-up. Implementation also requires standard operating procedures for patient preparation and image acquisition, staff training, documentation of excluded or low-quality images, predefined no-decision pathways for uncertain outputs, and local validation before routine use. From a regulatory and governance perspective, model updates, device changes, data protection, audit trails, performance drift, and post-deployment monitoring should be explicitly managed. These requirements support the use of thermography as a workflow-integrated adjunct rather than as an autonomous diagnostic decision-maker.

## 7. Conclusions

This review summarizes the current state and future directions of thermography-based artificial intelligence methods for evaluating arthropathies. Our synthesis indicates that the strongest current role of thermography is not as a standalone diagnostic replacement, but as a reproducible, low-burden functional imaging layer that can support screening, longitudinal monitoring, triage, and selection of patients who require confirmatory ultrasound or MRI. To bridge the gap between research and clinical utility, these frameworks call for strict reporting standards and reliable methodologies. Conforming to these standards will improve transparency, reduce bias, and increase the reproducibility of thermography-based AI models. These measures will support increased adoption of AI as diagnostic and prognostic tools in clinical rheumatology. Establishing a strong methodological structure will also facilitate the integration of advanced imaging techniques, particularly infrared thermography, into routine clinical care. Despite recent progress, significant difficulties remain in implementing AI models in clinical settings. Additional research is needed to standardize data collection, validate models among varied populations, and develop interpretable AI systems that build clinician confidence and encourage wider use.

## Figures and Tables

**Table 1:** Quantitative comparison of ThermoJIS, ThermoDAI, and ThermoDAI-CRP.

Study/Index	Intended Use	Dataset and Validation Cohort	Reference Standard	Input Data and Model	Main Quantitative Findings	Main Limitations	Clinical Implication
ThermoJIS [16]	Detection of active synovitis in RA	595 total participants; 449 development participants with RA, PsA, UA, secondary arthritis, OA or no rheumatic disease; 146 RA patients in validation set	Bilateral hand ultrasound using GS and PD scoring; active synovitis defined as GS sum score > 1 and PD sum score > 0	Hand thermograms; automated preprocessing; local thermal feature extraction; k-nearest-neighbors model; patient-level ThermoJIS	Correlation with GS ρ = 0.49 and PD ρ = 0.51; AUROC 0.78 for active synovitis; cutoff 3.56: sensitivity 94%, specificity 51%, PPV 68%, NPV 88%, F1-score 0.79; excluding indeterminate values 3.46–5.65: AUROC 0.86, sensitivity 87%, specificity 82%, PPV 81%, NPV 88%, F1-score 0.84, but 43% indeterminate	Internal validation only; need external validation; no tenosynovitis assessment; moderate correlation with ultrasound; lower specificity when used without indeterminate zone	Sensitive screening/triage tool for ultrasound-defined synovitis; useful for identifying possible subclinical inflammation but not sufficient as a stand-alone diagnostic test
ThermoDAI [54]	Remote or simplified RA disease activity assessment without formal joint counts	146 RA patients from ThermoJIS validation cohort	Ultrasound GS/PD scores; comparison with CDAI	ThermoJIS + PGA; simple linear composite score, range 0–20	Correlation with GS ρ = 0.52 and PD ρ = 0.56; strong correlation with CDAI ρ = 0.83; thresholds: remission 5.3, LDA 10.3, HDA 13.2; weighted kappa with CDAI 0.73; sensitivity 96% and specificity 30% for active synovitis using remission threshold	Cross-sectional design; low specificity; requires validation of longitudinal responsiveness and treatment-response thresholds; depends on ThermoJIS model stability	Promising remote monitoring adjunct when joint counts are unavailable; high sensitivity favors screening rather than definitive disease activity classification
ThermoDAI-CRP [54]	Remote or simplified RA disease activity assessment incorporating inflammatory biomarker	146 RA patients from ThermoJIS validation cohort	Ultrasound GS/PD scores; comparison with SDAI and DAS28-CRP	ThermoJIS + PGA + CRP; simple linear composite score, range 0–30	Correlation with GS ρ = 0.58 and PD ρ = 0.61; strong correlations with SDAI ρ = 0.85 and DAS28-CRP ρ = 0.81; thresholds: remission 6.4, LDA 10.9, HDA 14.1; weighted kappa with SDAI 0.73; sensitivity 88% and specificity 35% for active synovitis using remission threshold	Cross-sectional design; specificity remains low; requires CRP availability; CRP may be normal despite active synovitis or elevated for non-articular reasons	May improve remote RA assessment when CRP is available; should be interpreted with clinical context and confirmatory imaging when results are discordant

RA—rheumatoid arthritis, PsA—psoriatic arthritis, UA—undifferentiated arthritis, OA—osteoarthritis, GS—gray-scale ultrasound, PD—power Doppler ultrasound, ThermoJIS—thermographic joint inflammation score, k-NN—k-nearest neighbors, ρ—Spearman’s correlation coefficient, AUROC—area under the receiver operating characteristic curve, PPV—positive predictive value, NPV—negative predictive value, F1-score—harmonic mean of precision and recall, ThermoDAI—thermographic disease activity index, PGA—patient global assessment, CDAI—Clinical Disease Activity Index, LDA—low disease activity, HDA—high disease activity, CRP—C-reactive protein, ThermoDAI-CRP—thermographic disease activity index combined with C-reactive protein, SDAI—Simplified Disease Activity Index, DAS28-CRP—Disease Activity Score in 28 joints using C-reactive protein. Taken together, experience from radiomics and from RA thermography suggests that feature selection and dimensionality reduction should be treated as core steps in thermogram-based biomarker development, rather than afterthoughts. A pragmatic pipeline would (i) remove clearly unstable or redundant thermographic descriptors, (ii) apply supervised feature selection embedded in model training to retain only the most informative predictors, and (iii) when appropriate, use dimensionality reduction techniques to summarize correlated features into a small number of components. The end goal is to derive a few robust, interpretable indices, such as ThermoJIS and ThermoDAI-type scores, that can be validated externally and used alongside conventional clinical and imaging endpoints in the management of inflammatory arthropathies.

**Table 2:** Summative comparison of ROI definition strategies for thermographic assessment of arthropathies.

ROI Strategy	Reproducibility	Feasibility	Expertise Required	Robustness
Manual ROI selection	Moderate to high when performed by trained evaluators under standardized protocols; however, inter- and intra-rater variability remains important, especially for small joints or repeated measurements.	Feasible in single-center studies and small datasets, but time-consuming when many joints, visits, or thermograms are analyzed.	High anatomical and thermographic expertise; operators must consistently identify joint landmarks and avoid artifacts.	Limited robustness to posture changes, framing differences, and evaluator fatigue.
Rule-based ROI construction	Higher reproducibility than purely manual ROI when geometric rules, fixed dimensions, and anatomical assumptions are predefined.	Feasible when image acquisition is standardized and joint positioning is consistent.	Moderate expertise; requires protocol design and anatomical knowledge, but less manual interpretation during analysis.	Robust in controlled settings, but less reliable when anatomy, posture, or image framing varies.
Landmark-guided extraction	Good reproducibility when anatomical, fiducial, or detected landmarks provide stable spatial anchors for ROI placement.	Feasible in structured acquisition protocols; can support repeated measurements and longitudinal comparison.	Moderate to high expertise during protocol setup; lower expertise during routine use once landmarks are standardized or automatically detected.	More robust than simple rule-based ROIs because ROI placement is anchored to anatomical or external reference points.
Learning-based segmentation/keypoint detection	Potentially high reproducibility if trained and validated on representative datasets; performance should be reported with segmentation metrics such as Dice or IoU when reference masks are available.	Highly feasible for large datasets and AI pipelines after model development, but requires annotated data and computational resources.	High expertise during development, annotation, training, and validation; lower expertise during deployment if the model is integrated into software.	Potentially robust to anatomical and positional variability but sensitive to domain shift, camera differences, acquisition protocol changes, and dataset bias.

ROI—region of interest, AI—artificial intelligence, Dice—Dice similarity coefficient, IoU—intersection over union.

**Table 3:** Comparative overview of ML, DL, and feature-based computational studies for thermographic arthropathy assessment.

Study	Disease and Joint	Sample Size	Input and Method	Validation Design	Key Performance Metrics	Primary Purpose	Key Limitation
Morales-Ivorra et al. 2022 [16]	RA; bilateral hands	595 (449 training set, 146 validation)	Hand thermograms; k-NN composite (ThermoJIS)	Internal split-sample	AUROC 0.78; Sn 94%, Sp 51%; indeterminate zone: AUROC 0.86, Sn 87%, Sp 82%	Diagnostic accuracy	Internal only; see Table 1
Morales-Ivorra et al. 2024 [15]	RA; bilateral hands	77 RA; 3 hospitals; 12 weeks	Smartphone camera; pre-trained ThermoJIS/ThermoDAI/ThermoDAI-CRP	Prospective multi-center external validation	Confirmed longitudinal responsiveness; sensitivity to treatment-related change	Longitudinal monitoring and external validation	Small *n*; single camera
Triantafyllias et al. 2024 [45]	Mixed arthritis (RA, PsA, SpA, gout); multi-joint	75 patients + 70 controls; 360 + 1808 joints	Multi-joint thermograms; k-means hotspot/ROI ratio (HRR)	Single-center cross-sectional	Overall: AUC 0.76 (0.70–0.82), Sn 79%, Sp 65%; wrist: AUC 0.91 (0.84–0.98), Sn 83%, Sp 88%	Diagnostic accuracy vs. power Doppler US	Mixed disease spectrum; single center; no external validation
Zhao et al. 2022 [39]	Pediatric active arthritis (JIA); knee and ankle	Development + validation cohort; sizes not reported in abstract	Within-limb calibrated ΔT (TAWiC); ROC thresholds	Split development/validation cohort	Knee validation: Sn 0.60–0.70, Sp > 0.90 across views	Calibrated biomarker development	Pediatric only; clinical exam as validation reference (not US)
Tan and Lim 2025 [65]	RA; bilateral knees	95 RA; 570 thermograms; 190 knees	Manual ROI; T-min, T-max, T-avg at lateral, anterior, medial knee	Cross-sectional; ICC reliability subset	AUC 0.63–0.82 (PD > 0 and GS ≥ 2); ICC 0.997–0.999	Correlation and reproducibility	Manual ROI; single center; cross-sectional
Ahalya et al. 2023 [64]	RA vs. healthy controls; bilateral hands	240 thermal images (4 views/subject; subject *N* not stated)	k-means segmentation; BRISK/MSER/FAST/ORB features; LogitBoost, SVM, QSVM	Internal 10-fold CV and 80–20% split	LogitBoost: 93.75%; QSVM: 92.7%	Proof-of-concept feature-based classification	Small dataset; no external validation; no imaging reference; RA vs. healthy only
Ahalya et al. 2023 [76]	RA vs. healthy controls; bilateral hands	100 total (50 RA, 50 healthy)	Hand thermograms; RANet (custom CNN); ResNet101V2; InceptionResNetV2; DenseNet201; QNN	Internal cross-validation	RANet: 95%; RANet + SVM: 97%; QNN: 93.33%	Proof-of-concept DL classification	Small dataset; no external validation; no Sn/Sp vs. imaging reference; RA vs. healthy only
Kesavapillai et al. 2024 [58]	RA vs. healthy controls; bilateral hands	100 total (50 RA, 50 healthy)	Thermograms + radiographs; RA-XTNet (CNN-Transformer); UNet++; ViT; QSVM	Internal cross-validation	RA-XTNet (thermal): 93%; UNet++ IoU 0.87, Dice 0.86, pixel accuracy 98.75%; ViT: 90%; QSVM: 87.5%	Proof-of-concept DL classification and segmentation	Requires thermal AND radiographic input; small dataset; no external validation
Alarcón-Paredes et al. 2021 [46]	RA screening; women; bilateral hands	External test *N* = 38 (full training *n* not reported)	Thermal images + RGB photographs + grip force; random forest	Split-sample + external test set	AUC > 0.94 (thermal and RGB); 94.7% accuracy (external test)	Proof-of-concept multimodal screening	Requires three input modalities; women only; small external test; single center
Ávila-Camacho et al. 2025 [12]	Mixed arthritis/arthrosis vs. healthy; bilateral hands	100 total (70 arthritis/arthrosis, 30 healthy)	Portable prototype (Raspberry Pi 4 + thermal camera); ResNet50	Internal; controlled environment	Overall accuracy ~64%; sensitivity 100% for healthy detection	Engineering prototype feasibility	Low overall accuracy (64%); no disease subtyping; no external validation

RA—rheumatoid arthritis, PsA—psoriatic arthritis, SpA—spondyloarthritis, JIA—juvenile idiopathic arthritis, ROI—region of interest, k-NN—k-nearest neighbors, ThermoJIS—thermographic joint inflammation score, ThermoDAI—thermographic disease activity index, ThermoDAI-CRP—thermographic disease activity index combined with C-reactive protein, CRP—C-reactive protein, AUROC—area under the receiver operating characteristic curve, AUC—area under the curve, Sn—sensitivity, Sp—specificity, US—ultrasound, HRR—hotspot-to-region ratio, ΔT—temperature difference, TAWiC—temperature asymmetry within-calibrated thermography, ROC—receiver operating characteristic, T-min—minimum temperature, T-max—maximum temperature, T-avg—average temperature, PD—power Doppler, GS—gray-scale ultrasound, ICC—intraclass correlation coefficient, BRISK—binary robust invariant scalable keypoints, MSER—maximally stable extremal regions, FAST—features from accelerated segment test, ORB—oriented FAST and rotated BRIEF, SVM—support vector machine, QSVM—quantum support vector machine, CV—cross-validation, CNN—convolutional neural network, ResNet101V2—residual neural network 101 version 2, InceptionResNetV2—inception-residual convolutional neural network version 2, DenseNet201—densely connected convolutional network 201, QNN—quantum neural network, DL—deep learning, RA-XTNet—rheumatoid arthritis X-ray/thermal network, UNet++—nested U-Net architecture, ViT—vision transformer, IoU—intersection over union, Dice—Dice similarity coefficient, RGB—red-green-blue image format, random forest—random forest classifier, ResNet50—residual neural network 50.

**Table 4:** Effects of acquisition heterogeneity on thermography-based arthropathy assessment.

Acquisition Factor	Effect on Diagnostic Thresholds	Effect on AI Model Generalizability	Effect on Cross-Device Reproducibility
Ambient temperature and humidity	May shift absolute skin temperature values and alter apparent cutoff points, especially when thresholds are based on °C values rather than within-subject contrasts.	Can introduce site- or session-specific thermal baselines that models may learn instead of disease-related patterns.	Reduces comparability when environmental conditions are not standardized or reported.
Acclimatization time and prior activity	Insufficient acclimatization or recent activity may increase or decrease local temperature, changing baseline values and ΔT estimates.	Adds physiological noise and may reduce model performance when training and deployment protocols differ.	Limits reproducibility across studies using different preparation intervals or activity restrictions.
Camera distance and angle	Alters apparent ROI size, pixel density, and edge effects, which can influence mean, maximum, percentile, or hotspot metrics.	Changes spatial feature distribution and may reduce transferability of models trained on fixed-view protocols.	Reduces repeatability when positioning geometry differs between devices, operators, or centers.
Camera resolution and thermal sensitivity	Affects detection of small hotspots, gradients, and upper-tail temperature descriptors, potentially changing diagnostic cutoffs.	Models may learn device-specific image texture, noise, or resolution patterns rather than physiological signals.	High-end and low-cost cameras may not produce interchangeable temperature maps without calibration or recalibration.
Emissivity and calibration settings	Incorrect emissivity or calibration can systematically bias absolute temperature measurements and threshold-based interpretation.	Systematic measurement bias can be embedded into model features and reduce external performance.	Limits direct comparison across cameras and software platforms using different correction settings.
Patient positioning and joint exposure	Changes heat distribution, visible anatomy, and reference regions, affecting side-to-side ΔT and local contrast metrics.	Produces inconsistent ROI inputs and increases the risk of learning posture- or framing-related artifacts.	Reduces reproducibility across repeated visits, centers, and operators.
ROI definition and segmentation protocol	Different ROI geometry changes extracted temperature statistics, hotspot burden, and asymmetry indices.	Alters the model input space and may invalidate models trained on different ROI conventions.	Makes cross-study comparison difficult unless ROI landmarks, geometry, and segmentation rules are explicitly standardized.

AI—artificial intelligence, ROI—region of interest, ΔT—temperature difference.

**Table 5:** Representative quantitative and AI-related outputs reported in thermography-based arthropathy studies.

Study/Context	Data or ROI Focus	Analytical Approach	Reported Quantitative Output	Key Translational Limitation
Knee arthritis thermography [34]	Joint-level knee ROIs	ROI temperature statistics	Tmax, Tmin, and average temperature compared across clinically/imaging-defined groups	Single-center design and protocol-dependent thresholds limit transferability.
Within-leg calibrated pediatric arthritis algorithm [39]	Pediatric lower-limb thermograms	Ipsilateral reference calibration using mean and upper-tail ΔT features	Calibrated ΔT metrics improved discrimination compared with uncalibrated absolute temperatures	Requires age-, anatomy-, and device-appropriate calibration.
ThermoJIS/ThermoDAI/ThermoDAI-CRP [16,54]	Hand thermography in 146 patients with RA	Machine-learning composite thermographic indices combined with clinical/CRP variables	Fair-to-strong correlation with ultrasound inflammation scores (ρ ≈ 0.52–0.61) and strong correlation with disease activity measures (ρ > 0.81)	Feature redundancy, cohort specificity, and device/protocol dependence require external validation and recalibration.
External validation of ML thermographic indices [15]	Prospective longitudinal RA assessment	Validation of previously developed thermographic indices	Supported longitudinal assessment of thermographic disease activity indices	Performance should still be tested across sites, camera systems, and acquisition protocols.
ROI reproducibility studies [67,68,69]	Standardized upper-limb or muscle ROIs	Reliability and repeatability analysis	Reported ICC values of 0.82–1.00, ICC > 0.94 for mean skin temperature, SEM 0.19–0.23 °C, and smallest detectable change 0.52–0.64 °C	Reliability estimates depend on ROI geometry, operator protocol, and camera stability.
Automated ROI extraction and segmentation [43,56]	Hand or multi-region thermal images	Automated segmentation/region extraction	Extraction of 44 ROIs with strong agreement with manual evaluators and nearly tenfold reduction in processing time; Dice/IoU should be reported when masks are available	Segmentation failures and domain shift may propagate into thermographic biomarkers and model predictions.

ROI—region of interest, Tmax—maximum temperature, Tmin—minimum temperature, ΔT—temperature difference, ThermoJIS—thermographic joint inflammation score, ThermoDAI—thermographic disease activity index, ThermoDAI-CRP—thermographic disease activity index combined with C-reactive protein, RA—rheumatoid arthritis, ML—machine learning, CRP—C-reactive protein, ρ—Spearman’s correlation coefficient, ICC—intraclass correlation coefficient, SEM—standard error of measurement, Dice—Dice similarity coefficient, IoU—intersection over union.

## Data Availability

No new data were created or analyzed in this study. Data sharing is not applicable to this article.
